# Intra-Atlas Node Size Effects on Graph Metrics in fMRI Data: Implications for Alzheimer’s Disease and Cognitive Impairment

**DOI:** 10.3390/s24030814

**Published:** 2024-01-26

**Authors:** Sahithi Kolla, Haleh Falakshahi, Anees Abrol, Zening Fu, Vince D. Calhoun

**Affiliations:** 1 Tri-Institutional Center for Translational Research in Neuroimaging and Data Science (TReNDS), Georgia State University, Atlanta, GA 30303, USA; skolla@student.gsu.edu (S.K.); aabrol@gsu.edu (A.A.); zfu@gsu.edu (Z.F.); 2 Georgia Institute of Technology, Emory University, Atlanta, GA 30303, USA; 3 Department of Computer Science, Georgia State University, Atlanta, GA 30303, USA

**Keywords:** functional network connectivity, fMRI, graph metrics, node size, brain networks

## Abstract

Network neuroscience, a multidisciplinary field merging insights from neuroscience and network theory, offers a profound understanding of neural network intricacies. However, the impact of varying node sizes on computed graph metrics in neuroimaging data remains underexplored. This study addresses this gap by adopting a data-driven methodology to delineate functional nodes and assess their influence on graph metrics. Using the Neuromark framework, automated independent component analysis is applied to resting state fMRI data, capturing functional network connectivity (FNC) matrices. Global and local graph metrics reveal intricate connectivity patterns, emphasizing the need for nuanced analysis. Notably, node sizes, computed based on voxel counts, contribute to a novel metric termed ‘node-metric coupling’ (NMC). Correlations between graph metrics and node dimensions are consistently observed. The study extends its analysis to a dataset comprising Alzheimer’s disease, mild cognitive impairment, and control subjects, showcasing the potential of NMC as a biomarker for brain disorders. The two key outcomes underscore the interplay between node sizes and resultant graph metrics within a given atlas, shedding light on an often-overlooked source of variability. Additionally, the study highlights the utility of NMC as a valuable biomarker, emphasizing the necessity of accounting for node sizes in future neuroimaging investigations. This work contributes to refining comparative studies employing diverse atlases and advocates for thoughtful consideration of intra-atlas node size in shaping graph metrics, paving the way for more robust neuroimaging research.

## 1. Introduction

The human brain, a marvel of biological complexity, intricately weaves together a multitude of interconnected regions. For decades, researchers have been captivated by the profound challenge of unraveling the enigmatic workings of the mind. This pursuit has led us to the realm of brain networks, including regions as nodes within a graph, with their interconnections depicted as edges [1,2]. Leveraging graph theory, a mathematical framework originally designed for analyzing networks and relationships, has proven invaluable in deciphering the brain’s mysteries. Notably, it has emerged as an indispensable tool for decoding neuroimaging data, ushering in a new era of understanding [1,3,4,5].

As we venture deeper into the study of brain function, a pivotal question arises: what constitutes a ‘node’ within these intricate brain networks? Some researchers construct intricate graphs using tiny brain units known as ‘voxels’, a method that offers its own set of advantages. Conversely, many others opt for an alternative approach known as the atlas-based method [6,7] In this method, atlases predominantly partition voxels into regions of interest (ROIs).

A large number of brain atlases are currently being utilized. Most of these have inherent variability in the size of the delineated ROIs within a given atlas. That is, across widely utilized atlases, the size of these regions can exhibit substantial disparities. For instance, the ratio of the largest ROI volume to the smallest ROI volume has been observed to vary from a modest 3.8 [8] to an astonishing 45 [7], or even an unprecedented 443 [6]. This striking heterogeneity in ROI size among different atlases underscores a dimension of the atlas-based neuroimaging pipeline that has received limited scrutiny.

While substantial research efforts have been dedicated to developing and validating various brain atlases, a noticeable gap remains in our understanding of how the size of ROIs/nodes, when employed in graph-based analyses, impacts the computed graph metrics. Graph theory and network science heavily rely on the precise delineation and characterization of these nodes, providing a mathematical foundation for modeling the intricate interplay of brain regions and extracting meaningful insights from functional and structural connectivity data. Nevertheless, the influence of varying node sizes within the context of graph theory remains predominantly uncharted territory. Ref. [9] investigates the impact of spatial and temporal scales on the characterization of human brain networks, revealing strong correlations between the number and diversity of motifs. The findings emphasize the importance of spatial resolution in comparing characteristic node fingerprints derived from both topological and spatial network features in connectome analysis. Ref. [10] investigates hub identification in healthy aging subjects’ brain networks using four measurements and explores the impact of various parcellation schemes, revealing consistent hub identification within the same type of brain network. These studies along with others predominantly focus on overall size rather than the relative sizes within subjects. Consequently, while these studies involve subdividing existing parcellations, they do not delve into the examination of cross-correlation between the ROI and graph metrics.

This paper endeavors to bridge this gap by conducting a quantitative investigation of the influence of node size on computed graph metrics. Through our research, we aim to shed light on whether and to what extent the size of nodes affects the outcomes of graph-based analyses. By addressing this important but often overlooked aspect of brain network analysis, we seek to contribute to the refinement of neuroimaging studies, ensuring their accuracy and interpretability.

The remainder of the paper is organized as follows: In Section 2, we elaborate on our methodology, focusing on the exploration of the relationship between intra-atlas node sizes and computed graph metrics using resting-state fMRI data. Section 3 provides a detailed presentation of our findings, specifically addressing the correlations between the size of nodes in the graph network and the corresponding values of graph metrics. In Section 4, we conduct a thorough review of these results, emphasizing the implications for understanding the inner structural characteristics of individual brain regions. Finally, in Section 5, we draw conclusions based on our analyses, highlighting insights gained from the node-metric coupling (NMC) outcomes and the impact of variations among different subjects or groups, particularly those diagnosed with Alzheimer’s disease and mild cognitive impairment.

## 2. Materials and Methods

We investigated the relationship between intra-atlas node sizes and computed graph metrics. To ensure the temporal coherence of the included timecourses, we adopt a fully automated data-driven functional atlas based on independent component analysis (ICA). Subsequently, we construct a graph by computing cross-correlations among the ICA timecourses, a method known as functional network connectivity (FNC). The determination of strong node connections involves the application of a ‘proportional thresholding’ (PT) technique [9], wherein connections are retained based on a predefined PT%. To quantify the size of each component node, we perform voxel counting within each component and subject, considering only those voxels with values exceeding a specified threshold.

We proceed to assess the structure of the graph using a combination of global and local metrics. Firstly, we determine the degree of each node, representing the count of connections linked to that node. Additionally, we calculate the characteristic path length, which provides the average shortest path length between all pairs of nodes, as well as identifies densely interconnected clusters of brain regions. These clusters play a vital role in revealing the functional specialization within the network, showcasing areas where neural processing is distinctly segregated. These path lengths and cluster formations are considered global metrics. Moreover, we identify specific regions within the brain known as ‘hubs’ for each node in the network. Hubs are central nodes that are more likely to participate in numerous shortest paths within the network. To quantify this centrality, we employ the closeness centrality metric, which measures a node’s connectivity to all other nodes in the network. These node-specific graph metrics allow us to gain insights into the inner structural characteristics of individual brain regions.

To explore the relationship between the size of nodes in the graph network and the corresponding values of graph metrics, we employ statistical analysis. Specifically, we compute the Pearson correlation between these two variables. The outcome of this correlation analysis is referred to as ‘node-metric coupling’ (NMC). Additionally, we investigate whether variations among different subjects or groups might influence the NMC. To explore this aspect, we extend our analysis to include a dataset consisting of resting fMRI data collected from individuals diagnosed with Alzheimer’s disease and mild cognitive impairment.

In the following sections, we begin by providing an overview of the dataset employed, followed by an exposition of our node estimation methodology. Subsequently, we detail our approach for establishing connections between graph metrics and node size.

### 2.1. Data Information

In this study, we leveraged functional magnetic resonance imaging (fMRI) data obtained from the Alzheimer’s Disease Neuroimaging Initiative (ADNI) dataset. The ADNI studies (ADNI-1, ADNI-GO, ADNI-2, and ADNI-3) have overarching goals centered around advancing research in Alzheimer’s disease (AD) and related conditions. Its primary objectives encompass the development of improved and standardized methods for acquiring longitudinal, multi-site MRI and PET data from patients with AD, mild cognitive impairment (MCI), and elderly controls. ADNI seeks to establish a comprehensive and widely accessible data repository, documenting longitudinal changes in brain structure and metabolism, while concurrently collecting clinical cognitive and biomarker data to validate imaging surrogates.

ADNI-1 includes a total of 800 subjects from 50 sites in the United States and Canada, and all subjects had clinical/cognitive assessments and 1.5 T structural MRI at specified intervals (6 or 12 months) for 2–3 years. Approximately 25% of subjects had MRI at 3 Tesla. In ADNI-GO/ADNI-2 (2010–2016), imaging was conducted using 3T scanners, employing T1-weighted imaging parameters akin to those in ADNI-1. ADNI-2 includes 550 newly enrolled subjects and approximately 450–500 followed from the original ADNI study. Instead of the dual-echo T2-weighted image utilized in ADNI1, 2D FLAIR, and T2*-weighted imaging were introduced at all sites. Each imaging session included both fully sampled and accelerated T1-weighted images. Advanced imaging techniques were incorporated based on the scanner manufacturer, featuring diffusion imaging on GE scanners, resting-state functional MRI on Philips scanners, and arterial spin labeling on Siemens scanners. Imaging for ADNI 3 is exclusively conducted using 3T scanners. In ADNI-3, 1070–2000 total participants enrolled across three cohorts (700–800 follow-up from ADNI2, and 370–1200 newly enrolled). Almost all imaging sequences from ADNI2 have been revised and incorporated into the ADNI 3 protocol. Each series in each exam undergoes two levels of quality control to ensure compliance with the protocol parameters and to assess series-specific quality, including factors such as subject motion and anatomic coverage. All subjects have clinical/cognitive assessments supervised by the study physician to ensure accuracy. During the on-site visit, a consent form was signed by every subject screened. ADNI dataset is widely used in the literature and Alzheimer’s disease investigations. More details of the ADNI dataset can be found in [10].

In this study, we investigate our methods using participants who underwent imaging using 3.0 Tesla scanners. The fMRI dataset utilized herein encompasses a comprehensive cohort from ADNI studies (ADNI-1, ADNI-GO, ADNI-2, and ADNI-3) consisting of 213 scans diagnosed with Alzheimer’s disease, 368 with mild cognitive impairment, and 947 control. Detailed subject characteristics for each group are provided in Table 1. Using an SPM 12 pipeline, preprocessing steps included rigid body motion correction to address subject head motion, correction for slice timing, normalization to the standard MNI space using the EPI template, resampling to achieve 3 × 3 × 3 mm isotropic voxels, and applying Gaussian smoothing with a kernel of FWHM = 6 × 6 × 6 mm.

### 2.2. Data-Driven Node Estimation

We employed independent component analysis (ICA) to identify the nodes of a graph, which involves decomposing whole-brain fMRI data into intrinsic connectivity networks (ICNs). Each ICN represents a functional unit comprising voxels that exhibit strong correlations in spontaneous BOLD signal fluctuations. However, when using fully blind ICA to extract ICNs, different components can be identified across datasets, leading to challenges in replication and comparison. To address this issue, we utilized the automated Neuromark pipeline [11]. This approach applies spatially constrained ICA guided by reliable network templates. To create the Neuromark templates and identify robust ICNs, we performed spatial ICA on two large datasets (the Human Connectome Project [HCP] and Genomics Superstruct Project [GSP]) of typical controls, resulting in group-level components. We then matched independent components from the HCP and GSP datasets based on spatial similarity (correlation ≥ 0.4) and selected reproducible ICNs as network templates. Five neuroscience experts performed ICN identification and functional domain labeling. Further details on the Neuromark templates can be found in [11]. Subsequently, we used the network templates as prior information within a spatially constrained ICA algorithm to obtain spatial maps and time courses (TCs) for the fBIRN dataset. For this purpose, we applied the multivariate objective optimization independent component analysis with reference (MOO-ICAR) method, implemented in the GIFT toolbox, specifically the Neuromark_fMRI_1.0 template, from TReNDS (http://trendscenter.org/software/gift accessed on 15 December 2021). Our choice of MOO-ICAR was based on its demonstrated performance in previous studies [12,13].

### 2.3. Estimation of Edge and Local/Global Metrics

To elucidate the functional connectivity within the brain network previously computed, we executed the following procedures: (1) calculating a functional network connectivity (FNC) matrix, and (2) quantifying the FNC network through the calculation of graph metrics. FNC is derived by computing Pearson cross-correlation coefficients among the ICA time courses [14]. This process yields a matrix from which we calculate graph metrics. Next, we concentrated our analysis on a selection of commonly employed local graph metrics (node degree, participation coefficient, and closeness centrality) as well as global graph metrics (global efficiency, characteristic path length, and clustering coefficient) within the graph.

Local graph metrics are instrumental in gaining insights into the attributes and roles of individual nodes, as well as the nature of their connections within the network [15]. These metrics provide a detailed understanding of how each node contributes to the network. The degree of a node in a network refers to the number of edges (connections) that are directly linked to that node. In other words, it quantifies how many neighbors or connections a node has in the network. A node with a high degree is considered more central or influential within the network. The participation coefficient assesses the extent of intermodular connections of individual nodes within a network. Nodes with low participation coefficients, often referred to as provincial hubs, tend to be primarily connected within their own module, indicating modular segregation. In contrast, nodes with high participation coefficients, known as connector hubs, play a crucial role in facilitating intermodular integration by connecting multiple modules within the network. Closeness centrality is a network metric used to quantify how close or central a specific node is to all other nodes in a network. It is calculated based on the shortest path distances between the node of interest and all other nodes in the network. In essence, closeness centrality measures how quickly a node can interact with or reach all other nodes in the network. A node with a high closeness centrality is considered more central and influential because it can efficiently communicate with other nodes, serving as a bridge for the flow of information. Mathematically, closeness centrality is often expressed as the reciprocal of the sum of the shortest path distances from the node to all other nodes in the network. Nodes with higher closeness centrality values have shorter average distances to other nodes, indicating their greater proximity and connectivity within the network [16].

Global graph metrics are quantitative measures employed to assess the overall structural properties of a network or graph. They offer valuable insights into the organization, connectivity, and behavior of the network as a whole [17]. In the context of brain network analysis, these metrics play a pivotal role in comprehensively describing various aspects such as network shape, functional integration, and communication (correlation) between different brain regions. Global efficiency is a graph metric used to quantify how efficiently information or signals can be exchanged or transferred throughout an entire network. It provides insight into how well a network facilitates global communication between its nodes.

Mathematically, global efficiency is typically defined as the reciprocal of the average shortest path length between all pairs of nodes in the network. The characteristic path length is a graph metric that quantifies the average distance between all pairs of nodes in a network. It provides insight into how well-connected or compact a network is. Mathematically, the characteristic path length is calculated as the average of the shortest path lengths (the minimum number of edges needed to travel from one node to another) between all possible pairs of nodes in the network. In simpler terms, it represents the average number of steps it takes to move from one node to any other node in the network. The clustering coefficient is a graph metric that quantifies the degree to which nodes in a network tend to form clusters or tightly interconnected groups. It measures the local density of connections within a network, providing insight into the presence of cliques or communities [16].

Table 2 presents a summary of the formulas employed for both local and global graph metrics [15,17,18,19,20].

### 2.4. Quantification of Node Size

We determine node size using the individual component maps generated via the Neuromark pipeline. This process involves thresholding each component image, using a threshold set at 3.5 times the standard deviation of the image data. Subsequently, we tally the number of voxels in the component map that surpasses this threshold. This count is proportional to the volume of highly participating voxels within the specific brain network and serves as the basis for comparing the graph metrics. Notably, the maximum-to-minimum volume ratio for the Neuromark atlas stands at 14.2, slightly exceeding that of the Craddock atlas (3.8) [8], yet notably lower than that of the Glasser atlas (45) [7]

### 2.5. Node-Metric Coupling

We investigated the association between node size in functional connectivity and brain network metrics (i.e., NMC) by computing the correlation between each graph metric and node size for subjects in our dataset, which included individuals with Alzheimer’s disease, mild cognitive impairment, and control participants. The correlation coefficient is calculated as

$$r=\frac{\sum \left(x-\bar{x})(y-\bar{y}\right)}{\sqrt{\sum {\left(x-\bar{x}\right)}^{2}\sum {\left(y-\bar{y}\right)}^{2}}}$$

where $\bar{x}$ and $\bar{y}$ are the sample mean.

Additionally, we examined whether NMC varied across subjects and among different groups. To do so, we assessed the significance of correlations within each group and compared NMC between groups. We assessed the significance of our results using Bonferroni correction for multiple comparisons at a significance level of 0.05, considering a total of 6 graph metrics and 53 components.

## 3. Results

**Figure 1:**
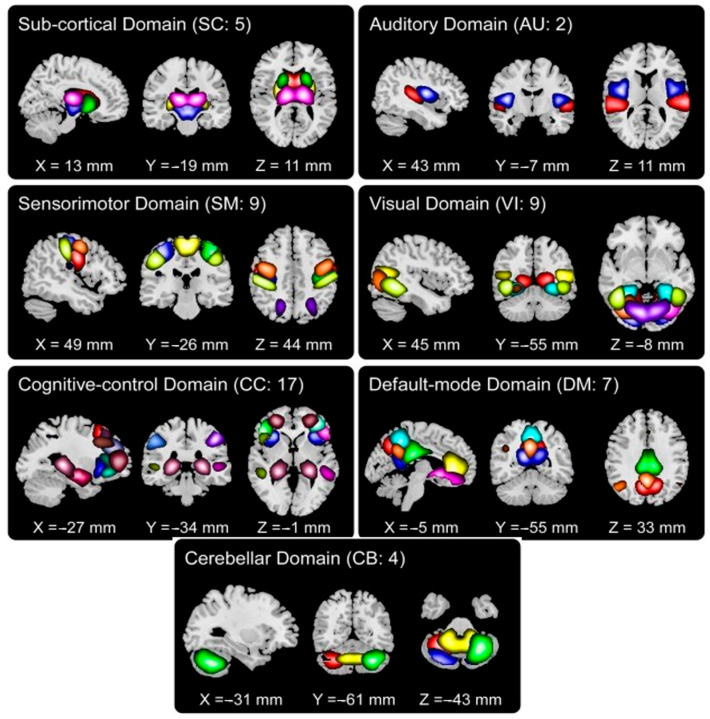
Fifty-three ICNs categorized into seven functional domains.

We chose 53 ICNs for subsequent analysis and grouped them into seven distinct functional domains. These labeled domains encompass auditory, sub-cortical, sensorimotor, visual, cognitive control, cerebellar, and default mode networks, as illustrated in Figure 1.

**Figure 2:**
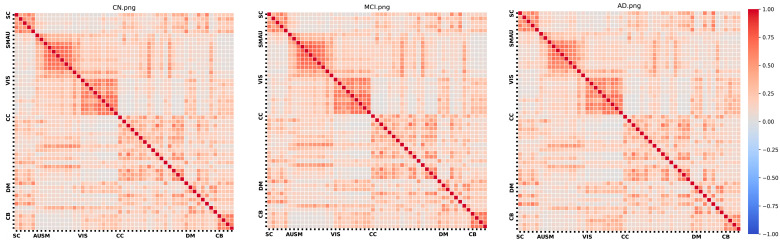
FNC matrices for each group.

Figure 2 presents the FNC matrix for each group, organized by functional domain. In the matrix, darker colors signify stronger positive or negative correlations, indicating robust relationships within the network. Conversely, lighter colors denote weaker relationships. Notably, the AD group has slightly, but significantly, lower FNC values than either the control or MCI groups.

**Figure 3:**
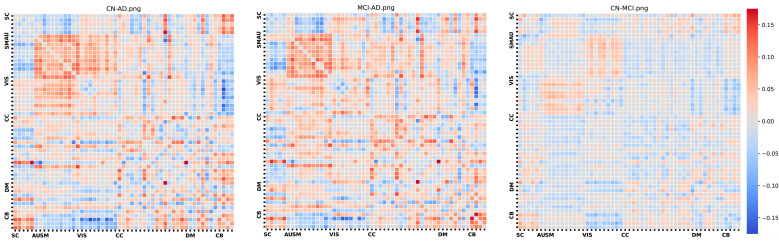
Group differences.

Figure 3 illustrates the group-wise differences in cellwise Functional Network Connectivity (FNC) between the AD, HC, and MCI groups. In this depiction, we note that the AD-HC comparison reveals elevated correlation values, indicating stronger connections among brain components, particularly within the subcortical, auditory, and visual domains. Within the cognitive control domain, there is a mixture of higher and lower correlation values. Furthermore, the AD group exhibits slightly weaker correlations, notably in the visual domain.

Table 3 presents group differences in global metrics, including global efficiency, characteristic path length, and clustering coefficient, among HC, MCI, and AD. When contrasting these metrics across different groups, we observe that both MCI and HC exhibit higher values for global efficiency compared to AD. Additionally, we assess brain network density using the clustering coefficient measure, revealing that node distances are lower in HC and AD when compared to MCI, along with similar findings for node density. Lastly, the segregation of nodes, as measured by the clustering coefficient, is lower in AD and HC relative to MCI.

Table 4 displays the outcomes of two-sample *t*-tests conducted to assess group differences in global metrics, including global efficiency, characteristic path length, and clustering coefficient, within the HC, MCI, and AD groups. All group comparisons yield significant results (marked with *), even after applying the False Discovery Rate (FDR) correction for multiple comparisons, except for the global efficiency in the HC-MCI comparison. This underscores the significance of evaluating node-metric coupling, as it demonstrates its significance even at the global level.

**Figure 4:**
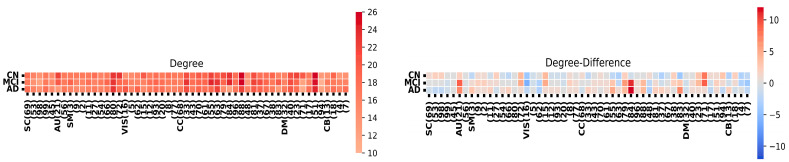
Node degree analysis and network differences among HC, MCI, and AD groups.

Figure 4 provides a component-level summary of the FNC matrix. We assess the significance of each node using three metrics: node degree, indicating nodes with numerous connections; closeness centrality, representing nodes in proximity to their neighbors; and participation coefficient, which gauges node activity across the network. Notably, the HC group exhibits a higher level of interconnectedness among nodes in the FNC matrix compared to both AD and MCI.

**Figure 5:**
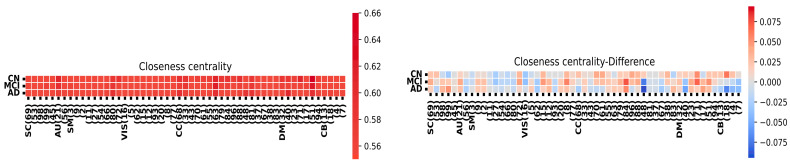
Closeness centrality analysis and network differences among HC, MCI, and AD groups.

Figure 5 provides a component-level summary of the FNC matrix, emphasizing the evaluation of the shortest pathways from one node to all others based on their closeness centrality. Closeness centrality is commonly defined as the normalized inverse of the total topological distances across the graph, often referred to as the nodes’ accessibility. Notably, in the FNC matrix, the HC group exhibits a greater level of integral connectivity among most nodes compared to MCI and AD.

**Figure 6:**
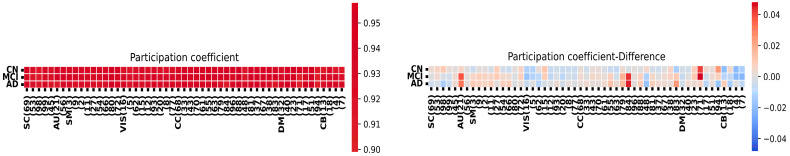
Participation coefficient analysis and network differences among HC, MCI, and AD groups.

In Figure 6, we present the results of the participation coefficient analysis in HC, MCI, and AD. The participation coefficient assesses how a node’s edges are distributed among the various communities within a graph. A node’s participation coefficient is zero if all its edges are exclusively connected to members of its own community. Notably, the figure reveals that after HC, AD exhibits the highest participation coefficient, while MCI shows the least difference in coefficients. We aim to identify the columns of significance among HC, MCI, and AD within the specified brain regions. Our goal is to discern whether AD and MCI exhibit significant values within these regions. Our observations indicate that MCI displays stronger significance within the considered regions compared to AD.

**Figure 7:**
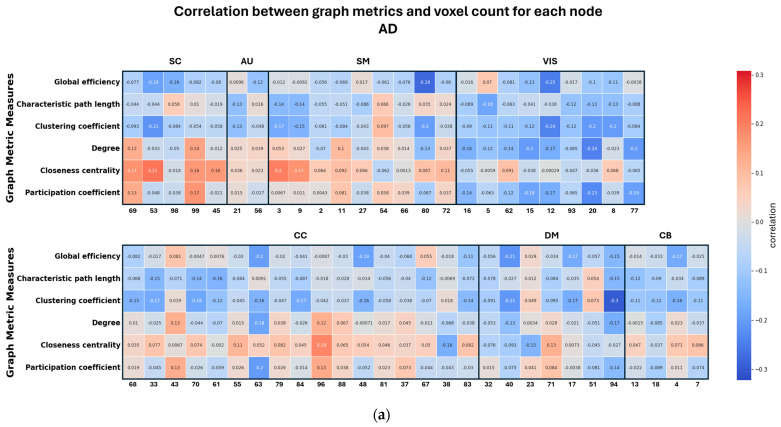

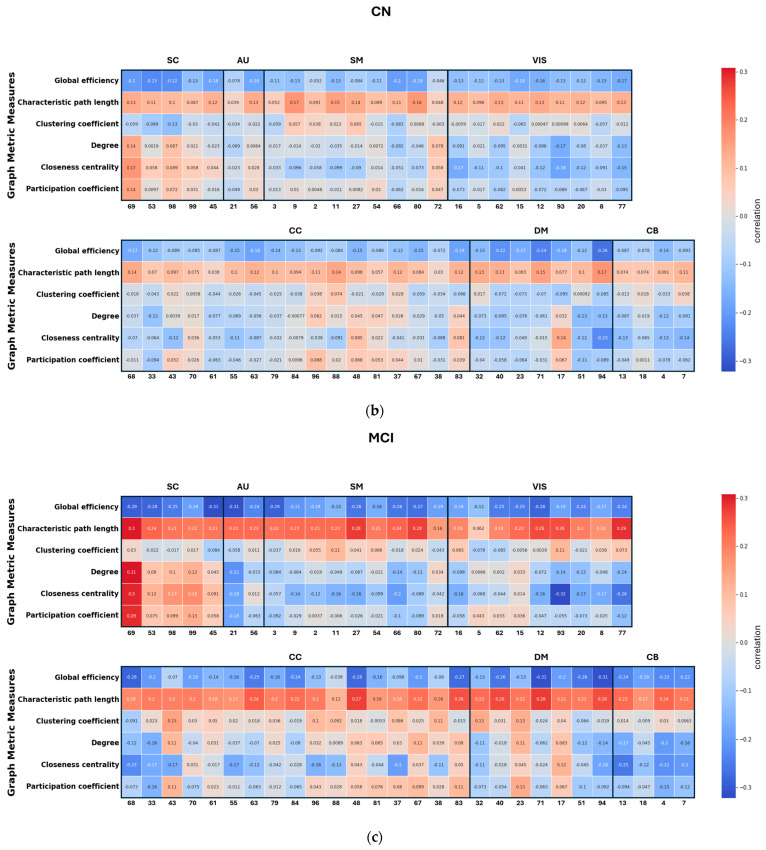
(**a**) Correlation between node size and the global and local metrics in the AD group. (**b**) Correlation between node size and the global and local metrics in HC. (**c**) Correlation between node size and the global and local metrics in MCI.

Figure 7 presents the key findings, emphasizing the relationship between graph metrics and node size within different brain network groups. In Figure 7a, it is evident that AD exhibits a robust correlation with global graph metrics compared to HC and MCI, while MCI displays a lower correlation with component graph metrics compared to AD and HC. These results underscore the highly significant relationship between node size and resulting graph metrics in each brain network. For instance, the characteristic path length shows a positive correlation in the control and MCI groups (meaning larger node size leads to greater characteristic path length), but a negative correlation in the AD group (larger node size leads to lower characteristic path length). Regarding global efficiency, coupling is predominantly negative in all three groups, indicating that larger nodes are associated with lower global efficiency. Other metrics, such as closeness centrality and participation coefficient, exhibit dependency on the specific network. For instance, subcortical networks display a positive node-metric coupling in all three groups, while most visual networks demonstrate a negative node-metric coupling. These differences may represent either a significant source of potential bias or a potential biomarker measure.

**Figure 8:**
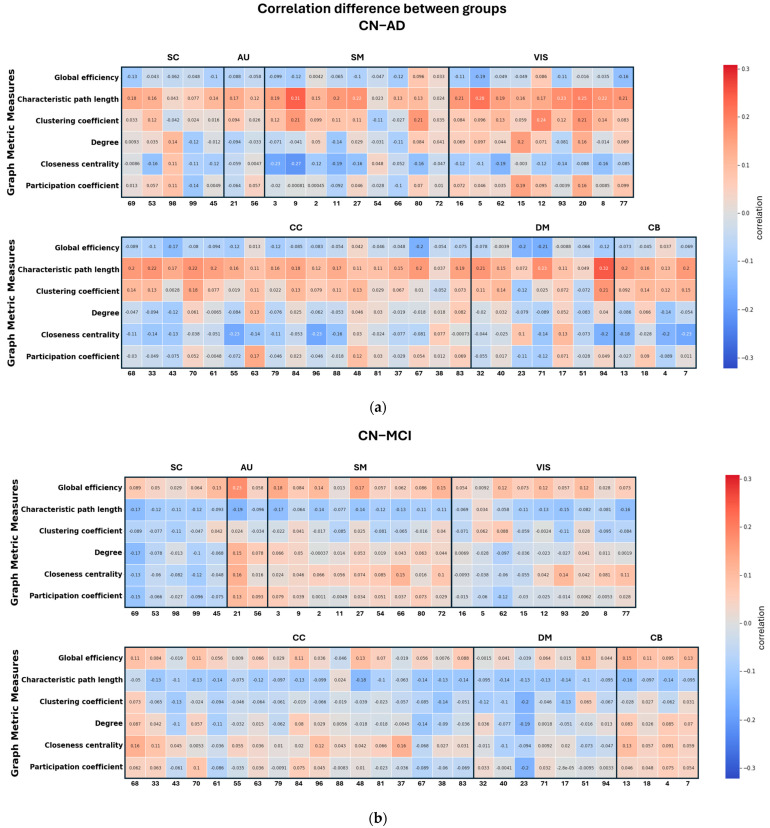

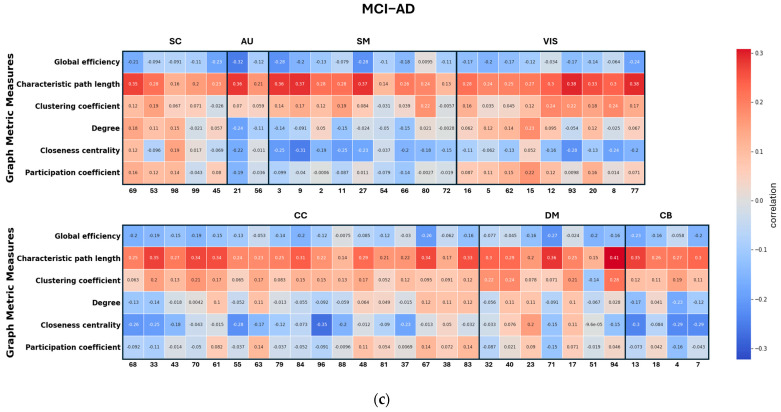
Differences in correlations between the global and local metrics. (**a**) AD-HC, (**b**) MCI-HC, (**c**) AD-MCI.

Figure 8 illustrates the differences in correlations between different groups. Notably, the AD group exhibits more pronounced differences in their correlations with graph metrics compared to both HC and MCI, whereas the differences between HC and MCI are comparatively smaller. This highlights the significance of the relationship between node size and graph metrics, which is also notably influenced by the specific graph metric under consideration.

Table 5 and Table 6 display the correlation values at which significance was achieved, along with the number of networks that exhibited significant correlation for each graph metric.

In general, our analysis reveals numerous significant differences in node-metric coupling across all tested graph metrics. Specifically, when examining the effects within the AD group, we observe that closeness centrality exhibits the most pronounced and significant differences. Meanwhile, characteristic path length and clustering coefficient show the most substantial differences when comparing AD with HC and MCI groups. Thus, the results underscore the substantial influence of node size on the resulting graph metrics.

## 4. Discussion

We conducted an initial study to explore the potential relationship between intera-atlas node size and resulting graph metrics. Our findings revealed a robust relationship, which also interacts with the comparisons between patients and controls. These results carry significant implications for the utilization of graph measures in neuroimaging research. Network science holds substantial promise for advancing our understanding of the human brain development, pathology, and aging, and such analyses are increasingly prevalent in the field [21]. Nevertheless, despite its widespread adoption, challenges persist in its application to neuroimaging data. One such challenge is the ongoing debate surrounding the definition of a network node in functional brain network analysis. An important caveat in previous studies lies in the absence of an accepted standard for defining brain nodes. The diverse array of approaches employed in this regard can significantly influence the outcomes. For instance, in the context of atlas-based node definition, which is commonly employed in most studies, researchers have the option to choose between anatomically predefined nodes, functionally informed nodes [22], or data-driven nodes [11,23]. However, more recent studies have begun to compare multiple atlases [24]. Notably, the published and shared atlases exhibit significant variations in the volumes of included brain regions. To date, there has been a lack of systematic research into whether the volume of brain regions included has an impact on the resulting graph metrics.

In this study, we adopted a data-driven approach to identify functionally defined nodes and investigate how their size influences the resulting metrics. We processed resting fMRI data via a fully automated ICA using the neuromark_fMRI_1.0 template. Subsequently, we applied a proportional threshold to prune node connections based on correlation, leading to the computation of FNC matrices, which represent the correlations among component-time courses. Based on these matrices, we derived various global and local graph metrics. Local metrics capture structural details at the component level, while global metrics provide an overall summary of the network structure. To assess the relationship between node size and graph metrics, we estimated node size by counting the number of voxels above a specified threshold. Larger node sizes corresponded to a higher voxel count.

Employing our node-metric coupling technique, we computed the cross-correlation between the obtained node sizes and graph metrics. To assess the impact of this relationship, we conducted a comparative analysis using a dataset comprising individuals from three categories: controls, individuals with mild cognitive impairment, and those diagnosed with Alzheimer’s disease. Our findings revealed a significant disparity in node-metric coupling across these groups. We observed that node size exhibited a substantial correlation with the resulting graph metrics. These two key findings suggest that node-metric coupling could potentially serve as a valuable biomarker for mental diseases. In summary, our findings underscore a robust connection between graph node size and graph metrics. Additionally, we identify noteworthy disparities in these correlations when comparing patient and control groups. This not only underscores the potential of our proposed joint approach, involving both node volume and graph metrics, as a potential brain-based marker but also emphasizes the necessity of meticulous evaluation when interpreting results in previous and forthcoming studies that rely on atlas-based methods employing nodes of irregular sizes. Interestingly, we observed variations in these correlations among different groups. Our findings specifically indicated that the Alzheimer’s group exhibited predominantly positive correlations between FNC and node properties compared to HC and MCI groups. This observation opens the possibility of considering NMC as a potential biomarker.

This study comes with some limitations. First, our initial assessment focused on only a subset of graph metrics, and there are numerous alternative approaches for characterizing a node’s role within a network. Some of these metrics operate at a local level, considering connections with neighboring nodes, while others operate at a global level, taking the node’s position within the entire network into account. Secondly, the dataset we utilized from the ADNI exhibited an imbalance between the number of Alzheimer’s and control subjects, with more data available for controls. This imbalance can lead to a higher likelihood of negative correlation values. To address this issue and enable meaningful comparisons across varying subject sizes, we implemented a proportional thresholding mechanism to eliminate low-impact areas of nodes within the dataset. It is worth noting that alternative thresholding strategies could be explored, and a more comprehensive investigation into the impact of thresholding on the results is a goal for future research. Additionally, this study centered on a single data-driven node parcellation approach. The choice of a data-driven atlas was deliberate, as it enables the grouping of temporally coherent and distinguishable brain regions, offering several advantages such as heightened sensitivity and the ability to capture dynamic changes within nodes [25]. In future research, it would be valuable to conduct a more extensive analysis encompassing various atlas-based approaches, each characterized by distinct node-size ratios. Furthermore, exploring the potential linkage between these changes and different types of Mild Cognitive Impairment (e.g., early MCI, late MCI, stable and progressive MCI) to determine if they hold predictive value for future cognitive decline could be an intriguing avenue for investigation. The deliberate use of multiple scans was intended to ensure a robust sample size and a comprehensive exploration of the method. Our analysis reveals a noteworthy observation—even when employing a subset of scans and considering only one scan per subject, our reported results demonstrate a remarkable level of consistency. This consistency not only reinforces the reliability of our findings but also suggests that the observed effects hold true, irrespective of the specific subset of scans or subjects considered. Furthermore, while our study has provided valuable insights into the relationship between intra-atlas node size and graph metrics, it is essential to consider the generalizability of our findings. The current investigation is based on the ADNI dataset and focuses on certain parameters. To enhance the generalizability of our results, future studies should consider diverse datasets and possibly different populations. Additionally, we acknowledge the need for replication studies to validate our findings across various contexts. As part of our ongoing research, we are committed to exploring these aspects to provide a more comprehensive understanding of the broader implications of our observations. Lastly, to address potential concerns related to the heterogeneity in data acquisition procedures and collection intervals, we conducted covariate analysis, revealing no significant impact on the study’s outcomes across different sites. It may be beneficial for future research to consider standardizing data collection intervals or employing additional statistical methods that account for such variations. This would contribute to a more consistent and rigorous approach, ensuring the integrity of the study’s findings.

In brief, our findings emphasize the substantial impact of node size on resulting graph metrics. This underscores both the challenge of comparing existing graph metric studies and the potential opportunity for node size to serve as an informative marker of brain health and disorders. Further research is essential to delve deeper into the node-metric coupling and its potential applications.

## 5. Conclusions

In the realm of neuroimaging research, it has been a common practice to disregard the influence of node size on graph metrics. However, our research has unveiled a compelling revelation: the size of a node plays a pivotal role in shaping the resulting graph metrics, and this interplay is not to be underestimated, particularly in the context of the statistical tests being employed. Our study introduces the concept of Node-Metric Coupling (NMC), shedding light on its potential to provide invaluable insights into the comparative analyses we undertake. Furthermore, NMC could potentially serve as a biomarker, highlighting its significance in understanding brain health and disorders. Looking ahead, future investigations should delve deeper into this intricate relationship. This exploration may involve conducting comparisons with voxel-level graphs, exploring the utility of uniformly sampled graphs, and conducting systematic assessments of the contrasts between atlas-based and data-driven approaches. These endeavors will contribute to a more comprehensive understanding of the dynamic interplay between node size and graph metrics in the context of neuroimaging studies.

## Figures and Tables

**Table 1:** Overview of analyzed participant demographics.

Group Categories	Number of Subjects	M/F Count		Average Subject Age
		Male	Female	
Healthy Controls (HC)	947	384	563	74
Mild Cognitive Impairment (MCI)	368	199	169	73
Alzheimer’s Disease (AD)	213	115	98	75

**Table 2:** Formulas for local and global graph metrics.

Graph Metrics	Formula
Node degree	$k_{i}^{w}\;=\;\sum_{j\in N}w_{ij}$ $w_{ij}$ represents the connection weights.
Participation coefficient	$y_{i}^{w}\;=\;1\;-\;\sum_{m\in M\;}{\left(\frac{k_{i}^{w}\left(m\right)}{k_{i}^{w}}\right)}^{2}$
Closeness centrality	${\left(L_{i}^{w}\right)}^{-1}\;=\;\frac{n-1}{\sum_{j\in N,\;j\neq i\;}d_{ij}^{w}}$
Global efficiency	$E^{w}\;=\frac{1}{n}\sum_{i\in N}\frac{\sum_{j\in N,\;j\neq i}\;{\left(d_{ij}^{w}\right)}^{-1}}{n-1}$ $d_{ij}^{w}\;is\;the$ shortest weighted path length between *i* and *j*
Characteristic path length	$L^{w}=\;\frac{1}{n}\sum_{i\in N}\frac{\sum_{j\in N,\;j\neq i}\;d_{ij}^{w}}{n-1}$
Clustering coefficient	$C^{w}\;=\;\frac{1}{n}\sum_{i\in N}\frac{2t_{i}^{w}}{k_{i}\left(k_{i}-1\right)}$ where the number of triangles is $t_{i}^{w}\;=\;\frac{1}{2}\sum_{j,h\in N}{\left(w_{ij}w_{ih}w_{jh}\right)}^{1/3}$ and $w_{ij}$ represents the connection weights.

**Table 3:** Global graph metric group differences ± standard error.

Graph Metric	HC-MCI	HC-AD	MCI-AD
Global Efficiency	0.007 ± 0.001	0.012 ± 0.001	0.005 ± 0.001
Characteristic Path Length	−1.505 ± 0.162	−1.6557 ± 0.207	0
Clustering Coefficient	−2.0458 ± 0.361	−0.4387 ± 0.566	0.0005 ± 0.0001

**Table 4:** Two-sample *t*-tests comparing group differences in global graph metrics.

Graph Metric	HC-MCI t-Value	HC-AD t-Value	MCI-AD t-Value
Global Efficiency	7.066	* 11.64	* 4.029
Characteristic Path Length	* −9.304	* −7.986	0.0
Clustering Coefficient	* −5.668	* −0.775	* 3.459

**Table 5:** The correlation values at which significance was achieved.

	Correlation
HC (significant value)	0.12
AD (significant value)	0.26
MCI (significant value)	0.20
MCI-CN	0.11
AD-CN	0.12
AD-MCI	0.16

**Table 6:** Number of networks that exhibited significant correlation for each graph metric.

Graph Metric	HC	AD	MCI	HC-MCI	AD-HC	AD-MCI
Global Efficiency	0	3	0	24	4	0
Characteristic Path Length	53	4	53	0	48	53
Clustering Coefficient	5	3	17	5	30	46
Degree	8	10	16	10	9	17
Closeness Centrality	9	26	8	12	3	5
Participation Coefficient	14	8	14	10	15	17

## Data Availability

The MRI data that support the findings of this work are available to researchers via the Alzheimer’s Disease Neuroimaging Initiative (ADNI) data access procedure described at http://adni.loni.usc.edu/data-samples/access-data/ accessed on 10 January 2024.
